# Functions of the bone morphogenetic protein signaling pathway through microRNAs (Review)

**DOI:** 10.3892/ijmm.2015.2060

**Published:** 2015-01-02

**Authors:** AKIKO HATA, HARA KANG

**Affiliations:** 1 Cardiovascular Research Institute, University of California at San Francisco, San Francisco, CA 94158, USA; 2 Division of Life Sciences, College of Life Sciences and Bioengineering, Incheon National University, Incheon 406-772, Republic of Korea

**Keywords:** bone morphogenetic protein, cell differentiation, microRNA

## Abstract

MicroRNAs (miRNAs or miRs) have emerged as key regulators of gene expression in essential cellular processes, such as cell growth, differentiation and development. Recent findings have established that the levels of miRNAs are modulated by cell signaling mechanisms, including the bone morphogenetic protein (BMP) signaling pathway. The BMP signaling pathway controls diverse cellular activities by modulating the levels of miRNAs, indicating the complexity of gene regulation by the BMP signaling pathway. The tight regulation of the levels of miRNAs is critical for maintaining normal physiological conditions, and dysregulated miRNA levels contribute to the development of diseases. In the present review, we discuss different insights (provided over the past decade) into the regulation of miRNAs governed by the BMP signaling pathway and the implications of this regulation on the understanding of the cellular differentiation of vascular smooth muscle cells (VSMCs), osteoblasts and neuronal cells.

## 1. Introduction

Bone morphogenetic proteins (BMPs) are members of the transforming growth factor (TGF)-β superfamily, acting as potent regulators during embryogenesis and controlling such events as vascular development, bone formation and neuronal differentiation (1). In response to the binding of BMP ligands, a membrane-bound heterotetrameric complex of type I and type II BMP receptors becomes activated. The active type II receptor kinase phosphorylates the type I receptor, which in turn activates the catalytic activity of the type I receptor. Consequently, the Smad signal transducers are phosphorylated, and the downstream signal is propagated. In the canonical signaling pathway, BMPs activate receptor-specific Smads (R-Smads), Smad1, Smad 5 and Smad 8. The phosphorylation of these R-Smads promotes their association with the common Smad (co-Smad), Smad4, in a complex that translocates to the nucleus and regulates gene transcription either positively or negatively (2). Recently, BMP signaling was demonstrated to directly control the processing of a cohort of miRNAs through the non-canonical role of R-Smads (3).

MicroRNAs (miRNAs or miRs) are small non-coding RNA molecules evolutionarily conserved from plants to humans (4). miRNAs are transcribed by RNA polymerase II as long primary transcripts known as pri-miRNAs, which encode either single or multiple miRNAs (5). Hairpin-structured pri-miRNAs are processed into 60–80 nucleotide (nt) precursor molecules (pre-miRNAs) by the p68-Drosha microprocessor complex (6). Pre-miRNAs are then exported from the nucleus to the cytoplasm by exportin 5. In the cytoplasm, the pre-miRNAs associate with Dicer, which cleaves the pre-miRNA into a miRNA (mature miRNA) approximately 18–24 nt in size (7,8). The miRNA duplex is then loaded onto Argonaute proteins and presented to the RNA-induced silencing complex (RISC) for the recognition of target mRNAs (9). The mature miRNA guides the RISC to partially complementary sequences within the target mRNAs to regulate target gene expression. The mature miRNAs generally repress protein-coding genes by promoting the degradation of mRNAs or repressing their translation (9). Individual miRNAs have tissue-specific or developmental stage-specific expression patterns and exhibit a broad range of roles in a wide range of developmental processes (10).

**Figure 1:**
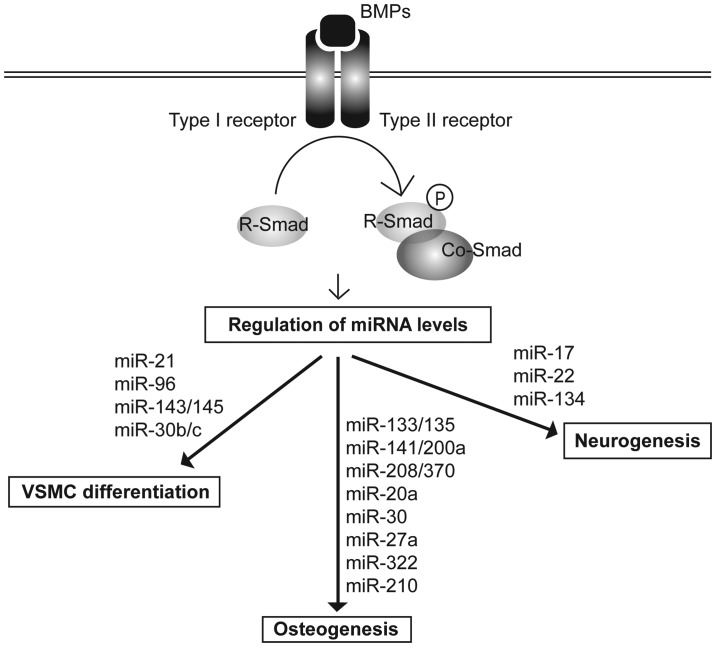
The bone morphogenetic protein (BMP) signaling pathway and the regulation of miRNA levels. BMP ligands induce the phosphorylation and translocation of Smads into the nucleus. The transcription of miRNAs or post-transcriptional miRNA processing is modulated by Smads. This regulation of miRNA levels is implicated in BMP signaling-mediated phenomena, such as vascular smooth muscle cell (VSMC) differentiation, osteogenesis and neurogenesis.

In the present review, we discuss and summarize the miRNAs that are mediated by the BMP signaling pathway in essential biological processes involving vascular smooth muscle cell (VSMC) differentiation, osteogenesis and neuronal development (Fig. 1).

## 2. VSMC differentiation

The inactivation of the BMP signaling pathway has been shown to result in the development of vascular disorders (11). For example, cells isolated from patients with heritable pulmonary artery hypertension (PAH) exhibit mutations of the type II BMP receptor (BMPRII) or Smad9 (12). Of note, although the loss of Smad9 function in the canonical BMP signaling pathway is largely compensated by Smad1 and Smad5, the mutation of Smad9 completely abrogates miRNA induction. This result suggests that the regulation of miRNAs by BMP signaling is implicated in normal vascular development and homeostasis. During vascular development, BMP signaling increases the expression of smooth muscle cell (SMC)-specific contractile genes and inhibits cell proliferation and migration, leading to the differentiation of VSMCs (13). The differentiated state of VSMCs is termed the ‘contractile phenotype’, and VSMCs can switch between the differentiated and dedifferentiated state in response to various environmental stimuli (14). Multiple miRNAs have been found to be regulated by BMP signaling and are responsible for VSMC differentiation and proliferation under physiological or pathological conditions.

### miRNA-21

Upon TGF-β and BMP signaling, Smads interact with p68 in the Drosha microprocessor complex and promote the cleavage of pri-miRNA-21 into pre-miRNA-21, leading to an increase in the levels of mature miRNA-21 in VSMCs (15). The increased miRNA-21 expression suppresses the expression of proteins, such as programmed cell death protein-4 (PDCD4) and multiple members of the dedicator of cytokinesis (DOCK) family, promoting the contractile phenotype of VSMCs (16).

In addition to miRNA-21, TGF-β and BMP signals modulate the expression of a subset of miRNAs through Smad-mediated post-transcriptional regulation (17). Notably, these miRNAs contain a conserved sequence (5′-CAGAC-3′) toward the center of the mature miRNA region that is identical to the consensus sequence for DNA binding by Smads.

### miRNA-96

The regulation of miRNA-96 expression by BMP signaling is critical for the modulation of the VSMC phenotype (18). miRNA-96 is downregulated by BMP4 in VSMCs, which results in the suppression of a novel target, Tribbles-like protein 3 (Trb3). Trb3 is an essential positive regulator of the BMP signaling pathway and promotes the contractile phenotype in VSMCs (19). The BMP-miRNA-96-mediated upregulation of Trb3 in VSMCs leads to an increase in SMC-specific gene expression. Unlike the regulation of miRNA-21 by BMP4, the downregulation of miRNA-96 by BMP4 is dependent on the signal transducer of the BMP signaling pathway, Smad4 (18).

### miRNA-302

BMP signaling also downregulates transcription of the miRNA-302~367 gene cluster in various types of cells, including VSMCs (20). This transcriptional repression of miRNA-302 by BMP signaling is mediated by Smads. Smad4 associates with the miRNA-302 promoter and recruits histone deacetylase (HDAC) to repress the transcription of miRNA-302. BMPRII has been identified as a novel target of miRNA-302. The functional consequence of the miRNA-302c-dependent downregulation of BMPRII on the BMP signaling pathway is the inhibition of the contractile phenotype of VSMCs. Therefore, the regulatory loop of BMP4-miRNA-302-BMPRII is an essential mechanism for the maintenance and fine-tuning of the BMP signaling pathway for the modulation of the VSMC phenotype (20).

### miRNA-143/145

miRNA-143 or miRNA-145 knockout mice exhibit an abnormal vascular tone and reduced SMC-specific gene expression in VSMCs, suggesting that miRNA-143 and miRNA-145, which are encoded as a gene cluster, play a critical role in the regulation of the VSMC phenotype (21). BMP signals activate the transcription of the miRNA-143/145 gene cluster through a consensus sequence termed the CArG box by serum response factor (SRF) and myocardin/myocardin-related transcription factor (MRTF)-A. miRNA-143/145 promote the contractile phenotype of VSMCs by regulating the expression of SMC-specific genes and cytoskeletal dynamics and by inhibiting the proliferation of VSMCs. miRNA-143/145 also repress multiple targets, including Kruppel-like factor 4 (KLF4), which is antagonistic to VSMC differentiation (22).

### miRNA-30b/c

miRNA-30b has been shown to be downregulated in human coronary artery atherosclerosis in calcified atherosclerotic vessels (23). An increase in BMP2 expression and a concomitant decrease in miRNA-30b expression were detected by *in situ* hybridization with vessels, suggesting that BMP signaling plays a role in VSMC calcification by regulating miRNAs. Indeed, a microarray analysis demonstrated that BMP2 decreases miRNA-30b and miRNA-30c expression, leading to the promotion of VSMC calcification (23). This downregulation of miRNA-30b and miRNA-30c is mediated by a Smad-independent pathway. Runt-related transcription factor 2 (Runx2) was identified as a target of miRNA-30b and miRNA-30c. Runx2 is a master transcription factor of the calcification process that induces the differentiation of osteoblasts and chondrocytes. The downregulation of miRNA-30b/c by BMP signaling is sufficient to increase Runx2 expression, which in turn results in the increased expression of the Runx2-dependent genes, osteopontin and osteocalcin, increased intracellular calcium deposition and the calcification of VSMCs (23).

## 3. Osteogenesis

Osteoblast differentiation is a key step in skeletal development, and precise control is necessary for the prevention of bone-related diseases. The activation of the TGF-β and BMP signaling pathways is involved in the differentiation of mesenchymal stem cells (MSCs) into the osteogenic lineage (24).

BMP2, 4 and 7 act as potential differentiators through the Smad-mediated activation of osteoblast essential genes, such as Runx2 (25). Recently, several miRNAs that are modulated by BMP signaling have been reported to regulate osteoblast differentiation either positively or negatively (26).

### miRNA-133/135

miRNA microarray analysis has revealed that miRNA-133 and miRNA-135 are downregulated during the BMP2-induced osteogenesis of C2C12 mesenchymal cells (27). Both miRNAs functionally inhibit the differentiation of osteoprogenitors by attenuating the Runx2 and Smad5 pathways. The BMP2-mediated downregulation of miRNA-133 is essential for the induction of Runx2 and osteogenic BMP2 signaling. The downregulation of miRNA-135 by BMP2 also permits BMP signaling through the derepression of its target, Smad5. Both Runx2 and Smad5 are essential for osteogenesis and synergize for the activation of bone-specific genes (28). Therefore, BMP2 controls bone cell determination by downregulating miRNA-133 and miRNA-135 expression, thereby releasing components required for osteogenic lineage commitment.

### miRNA-141/200a

miRNA expression in BMP-2-treated mouse pre-osteoblast MC3T3-E1 cells was previously investigated and the downregulation of miRNA-141 and miRNA-200a was observed (29). miRNA-141 and miRNA-200a modulate the BMP-2-stimulated pre-osteoblast differentiation. Transfection experiments with miRNA-141 or miRNA-200a have revealed the significant suppression of alkaline phosphatase (ALP) activity, which is widely accepted as a potential osteoblast differentiation marker. Both miRNA-141 and miRNA-200a target distal-less homeobox 5 (Dlx5). Dlx5 is an osteogenic transcriptional factor that modulates the expression of BMP2-induced osteogenic transcriptional master factors, such as Runx2 and Osterix (Osx) (30).

### miRNA-208/370

The expression levels of miRNA-208 and miRNA-370 have also been shown to be significantly decreased in BMP2- treated MC3T3-E1 cells (31,32). In cells transfected with miRNA-208 or miRNA-370, ALP activity and mineralization, as determined by Alizarin red staining, were suppressed. Moreover, the overexpression of miRNA-208 or miRNA-370 in primary murine osteoblast cells significantly attenuated BMP2-induced osteoblast differentiation (31,32). These results suggest that the downregulation of miRNA-208 and miRNA-370 is an important common phenomenon for osteoblast differentiation. miRNA-208 targets an osteogenic transcriptional factor, V-ets erythroblastosis virus E26 oncogene homolog 1 (Ets1). Ets1 activates the transcription of osteogenic genes, such as osteopontin (*OPN*), parathyroid hormone-related protein (*PTHrP*), *Runx2* and *tenascin-C* and *type I procollagen* (33). Furthermore, Ets1 is highly expressed during the proliferation stages in BMP2-treated MC3T3-E1 cells (33). Therefore, the enhanced expression of Ets1 through the downregulation of miRNA-208 and miRNA-370 upon BMP signals may be critical for osteoblast differentiation.

### miRNA-20a

miRNA-20a is a member of the miRNA-17–92 cluster, which is one of the most extensively studied families of miRNAs. The members of this family play important roles in tissue and organ development. During the course of osteogenic differentiation, the expression of endogenous miRNA-20a has been shown to be increased (34). Consistently, the transfection of miRNA-20a mimics or lentiviral miRNA-20a expression vectors into human MSCs promoted osteogenic differentiation. Notably, both the transcriptional and translational levels of BMP2, BMP4 and Runx2 were significantly elevated by miRNA-20a, but were decreased by anti-miRNA-20a (34). Moreover, miRNA-20a targets peroxisome proliferator-activated receptor γ (PPARγ), Bambi and Crim1, the negative regulators of BMP signaling (35). Therefore, miRNA-20a is an essential positive regulator that activates BMP signaling during osteogenic differentiation.

### miRNA-30

Emdogain is a clinical mixture of enamel matrix proteins that can induce biomineralization and osteogenesis (36). The expression profiles of miRNAs in MC3T3-E1 cells treated with Emdogain were previously investigated. The data indicated that the expression levels of miRNA-30 family members, such as miRNA-30a, -30b, -30c and -30d, were significantly downregulated during emdogain-induced osteoblast differentiation (37). miRNA-30a and miRNA-30d have been shown to be downregulated during the BMP2-induced osteogenesis of C2C12 mesenchymal cells as well (27), suggesting that the miRNA-30 family members function as negative regulators of osteoblastic differentiation. *Runx2* and *Smad1* were identified as common target genes of miRNA-30 family members (27). Smad1 is an immediate downstream transducing molecule of the BMP receptor and plays an important role in mediating BMP signaling (2). Therefore, the miRNA-30 family members affect osteogenesis by modulating BMP signaling.

### miRNA-27a

Special AT-rich sequence-binding protein 2 (Satb2) is a potent transcription factor that promotes osteoblast differentiation and bone regeneration. Satb2 functions as a protein scaffold to increase the activity of two essential osteogenic transcription factors, Runx2 and activating transcription factor 4 (ATF4) (38). The differentially expressed miRNAs induced by Satb2 overexpression in murine bone marrow stromal cells were previously investigated using miRNA microarray, and the downregulation of miRNA-27a was observed during osteoblast differentiation (39). miRNA-27a targets BMP2, bone morphogenetic protein receptor, type IA (BMPR1a) and Smad9, which are involved in the TGF-β/BMP signaling pathway (39). These results suggest that the negative regulatory role of miRNA-27a in Satb2-induced osteogenic differentiation is mediated by directly targeting positive regulators of the TGF-β/BMP signaling pathway.

### miRNA-322

miRNA-322 has been identified as a regulator of osteoblast differentiation (40). miRNA-322 gain- and loss-of-function experiments using C2C12, MC3T3-E1 cells and primary cultures of murine bone marrow-derived mesenchymal stem cells (BMMSCs) have demonstrated that miRNA-322 enhances the BMP2 response, increasing the expression of *Osx* and other osteogenic genes (40). The transducer of ERBB2 (Tob2) is characterized as a target of miRNA-322. Tob2 is a negative regulator of osteogenesis that binds and mediates the degradation of *Osx* mRNA (41). Therefore, miRNA-322 decreases *Tob2* mRNA and protein expression, leading to an increase in Osx expression. The lentivirus-mediated overexpression of miRNA-322 in BMMSCs repressed *Smad7,* as well as *Tob2* and induced *Osx* mRNA levels significantly (41).

### miRNA-210

The expression profiles of miRNAs during the osteoblastic differentiation of mouse ST2 mesenchymal stem cells were obtained by miRNA microarray analyses, and miRNA-210 was found to be highly expressed in these cells (42). Exogenous miRNA-210 positively regulates the osteoblastic differentiation of ST2 cells by targeting activin A receptor type 1B (AcvR1b). AcvR1b is a type I receptor known to transmit signals to R-Smad, Smad2 and 3, but not Smad1, 5 or 8, resulting in the transcription of genes that function as inhibitory regulators of cell proliferation (43). BMP signals, however, are transmitted through other receptors, such as AcvR1 (Alk2), BMPRIa (Alk3) and BMPRIb (Alk6), and their signals are transmitted to Smad1, 5 and 8, thereby initiating osteoblastic differentiation. Smad2/3 and Smad1/5/8 signaling has been reported to interfere with each other by competitive binding to a co-Smad, Smad4 (44). Therefore, miRNA-210 acts as a positive regulator of osteoblastic differentiation by inhibiting the Smad2/3 signaling pathway by targeting AcvR1b, resulting in the acceleration of Smad1/5/8-mediated osteoblastic differentiation.

## 4. Neurogenesis

In addition to well-characterized roles in bone development, BMP signaling is crucial during the development of the nervous system (45). BMP signaling is involved in the generation of the neural crest and the induction of both neuronal and glial fates from neural stem cells or neural precursors in the cortex, hippocampus, midbrain, hindbrain and spinal cord. By contrast, the inhibition of BMP signaling is required for the formation of the neural plate (45). The regulatory mechanism of BMP signaling is likely to be dependent on spatial and temporal factors, such as miRNAs. Indeed, the conditional knockout of Dicer in cortical neural progenitor cells (NPCs) impairs initial neuronal differentiation and later induces cell death, suggesting that miRNAs are necessary for appropriate cortical development or neuronal survival (46). Several miRNAs have been identified that can modulate the neural cell lineage during differentiation (47).

### miRNA-17

miRNA expression levels in the mouse cortex at different developmental stages have been investigated. The expression levels of miRNA-17 have been shown to be decreased in the developing cortex (48). As miRNA-17 represses the expression of BMPRII, the downregulation of miRNA-17 activates the BMP signaling pathway, which facilitates astrocytogenesis during differentiation (48). However, miRNA-17 promotes NPC proliferation, and the inhibition of BMP signaling contributes to miRNA-17-mediated increase of NPC proliferation (49). Therefore, miRNA-17 plays an important role during cortex development by modulating the BMP signaling pathway.

### miRNA-22

BMPs such as BMP2, 3 and 4 are expressed at the external germinal layer during postnatal cerebellum development and function as powerful inhibitors of sonic hedgehog (Shh)-mediated proliferation of cerebellar granular neuronal precursors (CGNPs) (50). To address whether the BMP signals that antagonize Shh-dependent proliferation are, at least in part, mediated by miRNAs, miRNA expression profiles in CGNPs in response to Shh were compared with those treated with Shh and BMP2 (51). miRNA-22 levels increased significantly following treatment with BMP2. miRNA-22 acts downstream of BMPs to modulate the activity of N-myc in CGNPs during the development of the cerebellum. The overexpression of miRNA-22 had a potent anti-proliferative effect, significantly increasing the cell cycle duration in CGNPs. Moreover, in P7 rat cerebellum, miRNA-22 distribution largely recapitulated the combination of BMP2 and BMP4 expression patterns (51). Therefore, BMP-mediated regulation of miRNA-22 is critical for neurogenesis by reducing the cell proliferation rate.

### miRNA-134

The expression of miRNA-134 has been shown to be increased during embryonic neuronal differentiation and modulates dendritic maturation in response to exogenous BMP4 by targeting the BMP antagonist, Chordin-like 1 (Chrdl-1) (52). The reduction in Chrdl-1 levels induced by miRNA-134 in dividing NPCs leads to the sensitization of cortical progenitors to autocrine BMP7 signaling, affecting NPC proliferation, neuron migration and neuronal maturation (52). Consistently, the sensitivity to exogenous BMP signals is reduced in miRNA-134-knockdown cells. Therefore, miRNA-134 is an essential mediator of BMP signaling-associated cortical development.

## 5. Conclusions

The BMP signaling pathway is involved in many cellular processes, including cell growth and differentiation. BMP signals either upregulate or downregulate a subset of miRNAs, and these coordinately regulated miRNAs cooperate for BMP signaling-mediated cellular functions. Therefore, it is important to understand how cells integrate the complicated regulation of miRNAs whose expression levels are fine-tuned by BMP signaling pathways and transmit a precise signal to control normal development and maintain homeostasis.

In the present review, we summarized the regulation of miRNA expression during BMP signaling pathway-mediated cellular differentiation, in particular VSMC differentiation, osteogenesis and neurogenesis. In most reported cases, miRNA levels are regulated by BMP signals, but BMP signaling is able to be regulated by miRNAs through targeting of BMP signal transducers or inhibitory molecules. For example, miRNA-30 and miRNA-133/135 target Smad1 and Smad5, respectively (27). This information provides insight into the mechansisms throug which miRNAs are integrated into the BMP signaling pathway and may help in the development of miRNA-based novel approaches to modulate the BMP signaling pathway for therapeutic applications.

Although the differential expression of a subset of miRNAs during BMP signaling-mediated differentiation has been elucidated in various contexts, many factors regulating the cellular miRNA levels are still unknown. Some miRNAs, such as miRNA-302, are transcriptionally regulated by direct binding of Smads to the promoter of the miRNA gene (20). Alternatively, other miRNAs are post-transcriptionally modulated by the association of Smads with pri-miRNA, such as miRNA-21 (15). Therefore, elucidating the mechanisms responsible for the regulation of miRNA remains a future challenge.
